# The Foxo1-YAP-Notch1 axis reprograms STING-mediated innate immunity in NASH progression

**DOI:** 10.1038/s12276-024-01280-5

**Published:** 2024-08-09

**Authors:** Dongwei Xu, Xiaoye Qu, Tao Yang, Mingwei Sheng, Xiyun Bian, Yongqiang Zhan, Yizhu Tian, Yuanbang Lin, Yuting Jin, Xiao Wang, Michael Ke, Longfeng Jiang, Changyong Li, Qiang Xia, Douglas G. Farmer, Bibo Ke

**Affiliations:** 1 The Dumont-UCLA Transplant Center, Division of Liver and Pancreas Transplantation, Department of Surgery, David Geffen School of Medicine at UCLA, Los Angeles, CA USA; 2 Department of Liver Surgery, Renji Hospital, Shanghai Jiaotong University School of Medicine, Shanghai, China

**Keywords:** Innate immunity, Metabolic disorders

## Abstract

Innate immune activation is critical for initiating hepatic inflammation during nonalcoholic steatohepatitis (NASH) progression. However, the mechanisms by which immunoregulatory molecules recognize lipogenic, fibrotic, and inflammatory signals remain unclear. Here, we show that high-fat diet (HFD)-induced oxidative stress activates Foxo1, YAP, and Notch1 signaling in hepatic macrophages. Macrophage Foxo1 deficiency (Foxo1^M-KO^) ameliorated hepatic inflammation, steatosis, and fibrosis, with reduced STING, TBK1, and NF-κB activation in HFD-challenged livers. However, Foxo1 and YAP double knockout (Foxo1/YAP^M-DKO^) or Foxo1 and Notch1 double knockout (Foxo1/Notch1^M-DKO^) promoted STING function and exacerbated HFD-induced liver injury. Interestingly, Foxo1^M-KO^ strongly reduced TGF-β1 release from palmitic acid (PA)- and oleic acid (OA)-stimulated Kupffer cells and decreased Col1α1, CCL2, and Timp1 expression but increased MMP1 expression in primary hepatic stellate cells (HSCs) after coculture with Kupffer cells. Notably, PA and OA challenge in Kupffer cells augmented LIMD1 and LATS1 colocalization and interaction, which induced YAP nuclear translocation. Foxo1^M-KO^ activated PGC-1α and increased nuclear YAP activity, modulating mitochondrial biogenesis. Using chromatin immunoprecipitation (ChIP) coupled with massively parallel sequencing (ChIP-Seq) and in situ RNA hybridization, we found that NICD colocalizes with YAP and targets *Mb21d1* (cGAS), while YAP functions as a novel coactivator of the NICD, which is crucial for reprogramming STING function in NASH progression. These findings highlight the importance of the macrophage Foxo1–YAP–Notch1 axis as a key molecular regulator that controls lipid metabolism, inflammation, and innate immunity in NASH.

## YAP-NICD interaction reprograms STING function in hepatic inflammation

In the battle against nonalcoholic steatohepatitis, it’s vital to understand how our immune system contributes to liver harm. Researchers found that a protein named STING is crucial in liver inflammation and damage as it identifies damaged DNA. They investigate how certain proteins and processes in immune cells affect STING’s function and NASH’s progression. Researchers discovered that decreasing the activity of a protein named Foxo1 in macrophagesresults in less liver damage and inflammation in mice on a high-fat diet. They also examined how other signaling processes, like the Hippo–YAP and Notch1 processes, interact with STING and contribute to the disease. Their findings indicate that adjusting these processes can reduce liver damage, steatosis, and inflammation, suggesting new potential treatment targets for NASH, potentially improving the lives of those affected by this condition.This summary was initially drafted using artificial intelligence, then revised and fact-checked by the author.

## Introduction

Innate immunity is essential for triggering hepatic inflammation in nonalcoholic steatohepatitis (NASH). Lipid accumulation in the liver induces lipotoxicity, which triggers cell death processes and releases damage-associated molecular patterns (DAMPs) that activate liver macrophages (Kupffer cells) and promote inflammation^1,2^. Mitochondrial DNA (mtDNA), a DAMP, has been shown to trigger specific immune responses and contribute to liver inflammation and injury^3^. Moreover, pathogen-associated molecular patterns (PAMPs), which are derived from microbial pathogens, such as bacterial lipopolysaccharide (LPS), RNA, and DNA, can reach the liver to activate innate immune cells, leading to increased hepatic injury in patients with NASH^4^.

Emerging evidence has indicated that lipid-induced oxidative stress involves activation of the endoplasmic reticulum (ER)-resident adaptor protein stimulator of IFN genes (STING)^5^. Indeed, STING is a cytosolic DNA sensor. Upon binding to DNA, the protein cyclic GMP-AMP synthase (cGAS) catalyzes the formation of cyclic GMP-AMP (cGAMP) from ATP and GTP. cGAMP binds and activates the adaptor protein STING to induce IRF3 activation via TANK-binding kinase 1 (TBK1)^6^. Lipid accumulation causes mitochondrial oxidative stress^7^, the release of mtDNA and the activation of STING^8,9^. STING deficiency reduces hepatocyte lipid accumulation and protects against alcohol-induced liver damage^5^. Moreover, STING is expressed in nonparenchymal liver cells, mainly macrophages or Kupffer cells, and disrupting STING expression in macrophages alleviated liver fibrosis and inflammatory responses^10,11^. Thus, these data suggest that STING is an essential adaptor protein that recognizes released DNA and triggers innate immune activation in NASH.

The FoxO subfamily of forkhead (Fox) transcription factors is a central player in cell differentiation, metabolism, and stress response^12^. Increasing Foxo1 activity promoted macrophage-mediated innate immune responses^13^. Oxidative stress augmented Foxo1 expression and activity in NASH patients^14^, suggesting that Foxo1 plays a pivotal role in regulating metabolic and immune homeostasis in the liver. Recently, innate immune activation was ultimately linked to the Hippo–Yes-associated protein (YAP) cascade^15^. YAP is modulated by its upstream kinases, mammalian Ste20-like kinase (MST)1/2 and large tumor suppressor (LATS)1/2, which phosphorylate YAP, resulting in cytoplasmic degradation^16^. However, unphosphorylated YAP enters the nucleus and interacts with various transcription factors to regulate gene expression^16^. The activation of YAP inhibits the immune response, whereas the disruption of YAP exacerbates tissue inflammatory injury^17^. Our previous studies showed that Hippo signaling regulated innate immunity via a YAP-mediated transcriptional mechanism^18^. These observations suggest that YAP functions as a suppressor of the innate immune response involved in inflammatory regulation.

In this study, we revealed a critical mechanistic link between the Foxo1-YAP-Notch1 axis and cGAS-STING-mediated innate immune responses in the development of NASH. High-fat diet (HFD)-induced oxidative stress activates macrophage Foxo1, YAP, and Notch1 signaling. Macrophage Foxo1 deficiency increased YAP activity and regulated lipid metabolism and mitochondrial functions by activating PGC-1α-mediated YAP activity. YAP directly interacts with the NICD, which is crucial for modulating its target gene *Mb21d1* (cGAS) and downstream effector STING, leading to reduced liver steatosis and inflammation.

## Materials and methods

### Animals

Floxed Foxo1 (Foxo1^FL/FL^, B6.129S6-*Foxo1*^*tm1Rdp*^/J), YAP (YAP^FL/FL^, B6.129P2-YAP1^tm1.1Dupa^/J), and Notch1 (Notch1^FL/FL^, B6.129×1-Notch1^tm2Rko^/GridJ) mice and mice expressing Cre recombinase under the control of the lysozyme 2 (Lyz2) promoter (LysM-Cre, B6.129P2-LYZ2^tm1(cre)Ifo^/J) were obtained from The Jackson Laboratory (Bar Harbor, ME). Myeloid-specific Foxo1, YAP or Notch1 knockout (Foxo1^M-KO^, YAP^M-KO^, and Notch1^M-KO^) mice were generated as previously described^18–20^. Myeloid Foxo1 and YAP double knockout (Foxo1/YAP^M-DKO^) and Foxo1 and Notch1 double-knockout (Foxo1/Notch1^M-DKO^) mice were generated by crossing Foxo1^M-KO^ mice with YAP^M-KO^ mice and Foxo1^M-KO^ mice with Notch1^M-KO^ mice, respectively. Mouse genotyping was performed using a standard protocol with primers described in the JAX Genotyping protocols database. Male mice aged 6–8 weeks were used in all experiments. This study was performed in accordance with the guidelines in the *Guide for the Care and Use of Laboratory Animals* published by the National Institutes of Health. Animal protocols were approved by the Institutional Animal Care and Use Committee of The University of California at Los Angeles.

### Mouse model of NASH

The WT, Foxo1, YAP, and Notch1 transgenic (Foxo1^FL/FL^, YAP^FL/FL^, and Notch1^FL/FL^) mice and myeloid-specific Foxo1, YAP, and Notch1 knockout (Foxo1^M-KO^, YAP^M-KO,^ and Notch1^M-KO^) or Foxo1/YAP or Foxo1/Notch1 double-knockout (Foxo1/YAP^M-DKO^ and Foxo1/Notch1^M-DKO^) mice on the C57BL/6J background were used. All animals were bred in-house in a pathogen-free facility and fed an HFD (18.1% protein, 61.6% fat, and 20.3% carbohydrates; D12492; Research Diets, New Brunswick, NJ) for 24 weeks starting at the age of 6 weeks. A methionine- and choline-deficient (MCD) diet (16.9% protein, 9.9% fat, and 64.9% carbohydrates; A02082002BR; Research Diets) was given to 6-week-old mice for 4 weeks. Mice fed a normal chow diet (NCD) (18.3% protein, 10.2% fat, and 71.5% carbohydrates; D12450B; Research Diets) served as controls.

### Liver lipid and function assays

Triglyceride (TG) and total cholesterol (TC) levels were measured in liver samples by commercial kits (Cayman Chemical and MyBioSource). Serum alanine aminotransferase (sALT) levels were measured by an ALT kit (Thermo Fisher) according to the manufacturer’s instructions.

### Histology, immunohistochemistry, and immunofluorescence staining

Liver sections were stained with hematoxylin–eosin (H&E), Oil Red O, Sirius Red, and Masson staining. Intracellular lipid droplets in primary hepatocytes were stained with Oil Red O. Liver macrophages were assessed using primary CD11b^+^ rat monoclonal antibodies (mAbs) (Abcam) and secondary Alexa Fluor 488- or Cy5-conjugated AffiniPure donkey anti-rat IgG (Jackson ImmunoResearch) for IF staining. DAPI was used for nuclear counterstaining. α-SMA was stained by IHC and IF with a primary mouse α-SMA mAb (Cell Signaling Technology). Double immunofluorescence staining of Foxo1, LIMD1, and LATS1 in liver sections and Kupffer cells was performed using primary Foxo1 mouse mAb (Cell Signaling Technology), CD68 rat mAb (Bio-Rad), LIMD1 mouse mAb (Santa Cruz Biotechnology), and LATS1 rabbit Ab (Thermo Fisher Scientific). Nuclear YAP and NICD were stained with the primary YAP rabbit mAb (Cell Signaling Technology) and the NICD mouse mAb (Santa Cruz Biotechnology). Images for immunofluorescence staining were captured using a fluorescence microscope and analyzed using Image-Pro Plus software. Positive cells were counted in 10 HPF/section (x200) in a blinded manner.

### Quantitative RT‒PCR analysis and mitochondrial DNA levels

Quantitative real-time PCR was performed using QuantStudio 3 (Thermo Fisher Scientific). The sequences of primers used for the amplification of TNF-α, IL-1β, IL-6, CXCL-10, Srebp1c, Fabp1, CD36, Fas, Accα, Cpt1α, Slc27a1, Acox1, Acadm, Abca1, Abcg1, Ldlr, Msr1, Hmgcr, Hmgcs, Gck, Pfk, Pklr, α-SMA, Col1α1, CCL2, Timp1, MMP1, TGF-β1, mtDNA, and GAPDH are shown in Supplementary Table 1. Target gene expression levels were calculated by their ratios to those of the housekeeping gene HPRT.

Total DNA from hepatocytes was obtained using TriPure® Isolation Reagent (Roche Diagnostics). Real-time PCR was performed as described above. The mitochondrial gene NADH dehydrogenase subunit ND4 was used for the semiquantification of mtDNA, and the nuclear gene GAPDH was used for normalization.

### Western blot analysis

Protein was extracted from liver tissue or cell cultures as previously described^18^. The nuclear and cytosolic fractions were prepared with NE-PER Nuclear and Cytoplasmic Extraction Reagents (Thermo Fisher Scientific). The Foxo1, p-JNK, JNK, cGAS, p-STING, STING, p-TBK1, TBK1, p-P65, P65, p-LATS1, LATS1, p-YAP, YAP, TFAM, COX-1, UCP3, Lamin B2, β-actin (Cell Signaling Technology), PGC-1α, LIMD1, and NICD (Santa Cruz Biotechnology) mAbs were used. The membranes were incubated with Abs, and a Western ECL substrate mixture (Bio-Rad) was then added for imaging with an iBright FL1000 (Thermo Fisher Scientific). Relative quantities of protein were determined by comparison to β-actin or Lamin B2 expression using iBright image analysis software (Thermo Fisher Scientific).

### RNA sequencing and bioinformatic analyses

Total RNA was extracted from liver macrophages from Foxo1^FL/FL^ and Foxo1^M-KO^ mice after 24 weeks of HFD feeding via the mirVana™ miRNA ISOlation Kit (Ambion-1561, Thermo Fisher Scientific) according to the manufacturer’s protocol. The quality of the samples was monitored with a NanoDrop ND-2000 system (Thermo Fisher Scientific) and an Agilent Bioanalyzer 2100 (Agilent Technologies, Santa Clara, CA). The samples with an RNA integrity number (RIN) ≥ 8 (7 ≤ RIN) were subjected to subsequent analysis. A total of 3 μg of RNA per sample was used as input material for the RNA sample preparations. Ribosomal RNA was removed by TruSeq Stranded Total RNA with a Ribo-Zero Golbin kit (RS-122-2301, Illumina, San Diego, CA), and rRNA-free residue was removed by RNAClean XP (A63987, Beckman Coulter, Indianapolis, IN). Sequencing libraries were generated using rRNA-depleted RNA via the NEBNext® Ultra™ Directional RNA Library Prep Kit for Illumina® (NEB, Ipswich, MA) according to the manufacturer’s instructions. Clustering of the index-coded samples was performed on a cBot Cluster Generation System using the TruSeq PE Cluster Kit v3-cBot-HS (Illumina) following the manufacturer’s recommendations. After cluster generation, the libraries were sequenced on an Illumina HiSeq 4000 platform, generating 150 bp paired-end reads. The raw fastq files were first trimmed to remove adaptors using Trim Galore (v0.5.0) with the following parameters: -q 25 -phred33 -length 35 -e 0.1 -stringency 4. Trimmed fastq files were then mapped to the grch38 or grcm39_tran genome utilizing hisat2 (v2.2.0)^21^. Gene expression was quantified using Cuffdiff (v2.1.1). Differentially expressed genes (DEGs) were analyzed by the edgeR R package (v3.18.1) using raw counts. An adjusted *P* value of less than 0.05 was set as the threshold to define DEGs. KEGG and GO analyses were performed using the Database for Annotation, Visualization, and Integrated Discovery (DAVID; version 6.8) and the R package “clusterProfiler” (v3.11.0).

### Isolation of hepatocytes, HSCs, and liver macrophages

Primary hepatocytes and liver macrophages (Kupffer cells) from Foxo1^FL/FL^, Foxo1^M-KO^, and Foxo1/Notch1^M-DKO^ mice were isolated as previously described^20^. In brief, the mice were anesthetized, the abdominal walls were opened, and the liver was perfused through the portal vein in situ with warmed (37 °C) EGTA-containing HBSS solution for 5 min, followed by collagenase-containing GBSS buffer (collagenase type IV, Sigma-Aldrich) for 5 min until liver digestion was visible. The perfused livers were dissected and passed through 70-μm nylon mesh cell strainers (BD Biosciences, San Jose, CA). Nonparenchymal cells (NPCs) were separated from hepatocytes by centrifugation at 50 × *g* for 2 min three times. NPCs were suspended in HBSS, layered onto a 50%/25% two-step Percoll gradient (Sigma) in a 50-ml conical centrifuge tube, and centrifuged at 1800 × *g* at 4 °C for 15 min. Kupffer cells in the middle layer were collected and allowed to attach to cell culture plates in DMEM supplemented with 10% FBS, 10 mM HEPES, 2 mM GlutaMax, 100 U/ml penicillin, and 100 μg/ml streptomycin for 15 min at 37 °C.

Primary hepatic stellate cell (HSC) isolation was performed with some modifications, as previously described^22^. Livers were perfused with EGTA, pronase, and collagenase solution. After centrifugation, the cell pellets were resuspended in density gradient medium. HSCs were identified as a white cell layer floating at the surface of the gradient. The HSC-containing cell layer was collected and cultured in HSC culture medium.

### BMM isolation and in vitro transfection

Murine bone-derived macrophages (BMMs) were generated as previously described^20^. In brief, bone marrow cells were collected from the femurs and tibias of mice by flushing the bone cavity with sterile cold Dulbecco’s phosphate-buffered saline without calcium or magnesium (Thermo Fisher Scientific). After centrifugation, the cell pellet was resuspended and cultured in DMEM supplemented with 10% FCS and 15% L929-conditioned medium at 37 °C in a 5% CO_2_ incubator. BMMs (1 × 10^6^ cells/well) from WT, Foxo1^FL/FL^, Foxo1^M-KO^, and Foxo1/Notch1^M-DKO^ mice were cultured for seven days and then transfected with CRISPR/Cas9-LIMD1 knockout (KO), CRISPR/Cas9-PGC-1α KO, CRISPR/Cas9-cGAS KO, and CRISPR/Cas9-YAP-KO or control vectors (Santa Cruz Biotechnology). After 24 h, the cells were incubated in cell culture medium supplemented with 0.2 mM palmitic acid (PA) and 0.4 mM oleic acid (OA) in 0.5% BSA for an additional 24 h.

### Coculture of macrophages and hepatocytes or HSCs

BMMs (1 × 10^6^ cells/well) isolated from Foxo1^FL/FL^, Foxo1^M-KO^, and Foxo1/Notch1^M-DKO^ mice were cultured in a 0.4 μm-pore size Transwell insert (Sigma-Aldrich) and transfected with the CRISPR/Cas9-YAP KO, CRISPR/Cas9-cGAS KO or control vector followed by PA and OA stimulation. Primary hepatocytes or HSCs (4 × 10^5^ cells/well) were cultured in a six-well plate. After 24 h, the Transwell insert containing BMMs was placed into a six-well plate, where hepatocytes or HSCs were initially seeded. The cocultures were incubated for 24 h.

### ELISA

Cell culture supernatants were harvested for cytokine analysis. The TGF-β1 levels were determined by an ELISA kit (Thermo Fisher Scientific).

### Immunoprecipitation analysis

BMMs from cocultures were lysed in NP-40 lysis buffer (Thermo Fisher Scientific). The lysates were incubated with NICD and YAP antibodies (Cell Signaling Technology) or with control IgG and protein A/G beads at 4 °C overnight. The immunocomplexes were analyzed using standard immunoblot procedures.

### Chromatin immunoprecipitation (ChIP)

ChIP analysis was carried out using a ChIP Assay Kit (Abcam) according to the manufacturer’s instructions. The sheared chromatin was immunoprecipitated with an anti-NICD or anti-YAP antibody overnight. Normal IgG was used as a control. For sequential ChIP, sheared chromatin was first immunoprecipitated with a YAP antibody, followed by a second immunoprecipitation with an NICD antibody. DNA from each immunoprecipitation reaction was examined by PCR. The primers for the NICD-responsive region of the cGAS promoter were as follows: forward, 5’-TTCTGCAAAGTAGGCAGCGT-3’; reverse, 5’- AACTTGTCTAACAAGCATTCGCT-3’.

### ChIP-sequencing (ChIP-seq)

The ChIP-DNA was amplified to generate a library for sequencing. Sequencing was performed on an Illumina HiSeq3000 (Illumina, San Diego, CA) for a single-read 50 run at the UCLA Technology Center for Genomics & Bioinformatics (TCGB). A data quality analysis was performed on an Illumina SAV. Demultiplexing was performed with the Illumina Bcl2fastq2 v2.17 program. Reads were mapped to the mouse mm10 genome using Bowtie1, and MACS2 was used for peak calling. ChIPseeker was used for peak annotation. Genome browser representation files were generated by converting ChIP-seq data to BigWig format.

### RNA in situ hybridization

RNA in situ hybridization (ISH) was performed using the RNAscope 2.5 HD Assay-RED Kit (324510, Advanced Cell Diagnostics) according to the manufacturer’s instructions. Mouse cGAS (Mm-cGAS-C1, 887941-C1) and negative and positive control probes were purchased from Advanced Cell Diagnostics. Fast Red was used to detect the RNA signal, and hematoxylin was used for counterstaining.

### Reactive oxygen species (ROS) assay

ROS production in hepatocytes was measured using 5-(and-6)-carboxy-2’,7’-difluorodihydrofluorescein diacetate (carboxy-H2DFFDA, Life Technologies) according to the manufacturer’s instructions. Positive green fluorescent-labeled cells were counted blindly at 10 HPF/section (×200).

### Statistical analysis

The data are expressed as the mean ± SD and were analyzed using the permutation *t* test and Pearson correlation. For each comparison, two-sided *P* values less than 0.05 were considered to indicate statistical significance. Multiple group comparisons were made using one-way ANOVA followed by Bonferroni post hoc correction. When groups showed unequal variances, we applied Welch’s ANOVA for various group comparisons. All analyses were performed with SAS/STAT software, version 9.4.

## Results

### Macrophage Foxo1 deficiency reduces hepatic steatosis and inflammation in response to HFD challenge

**Fig. 1:**
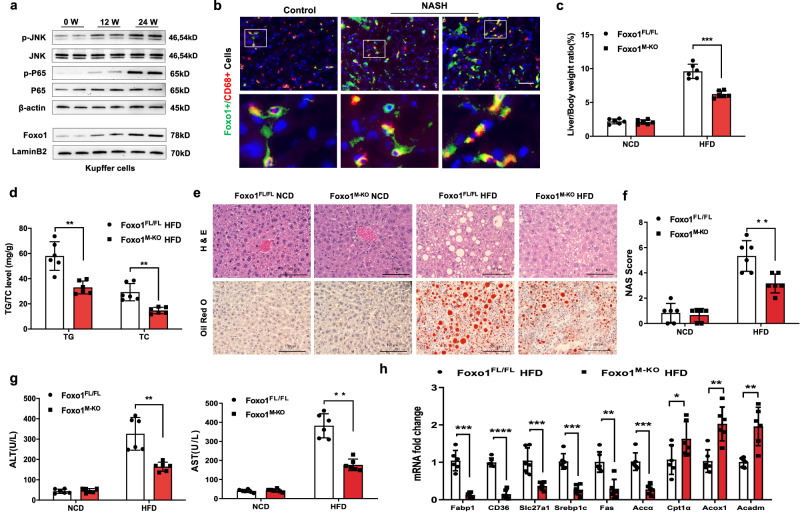
Macrophage Foxo1 deficiency reduces hepatic steatosis and inflammation in HFD-induced NASH. **a** The protein expression of nuclear Foxo1, p-JNK, JNK, p-P65, and P65 was upregulated in liver macrophages from wild-type (WT) mice after they were fed a high-fat diet (HFD) for 24 weeks, as determined by western blot analysis. The data are representative of three experiments. *Notes:* After 12 weeks of HFD feeding, nuclear Foxo1 expression was substantially increased in Kupffer cells. **b** Immunofluorescence staining showed that macrophage Foxo1 expression was increased in steatotic livers (*n* = 6 mice/group). Scale bars, 100 μm, and 30 μm. **c** The Foxo1^M-KO^ mice displayed lower liver-to-body weight ratios (*n* = 6 samples/group). **d** The levels of liver TG and TC (mg/g) in the Foxo1^M-KO^ livers were reduced (*n* = 6 samples/group). **e** Representative histological staining (H&E and Oil Red O) revealed that Foxo1^M-KO^ alleviated hepatic steatosis and reduced hepatocyte ballooning and lipid accumulation in the liver (*n* = 6 mice/group). Scale bars, 100 μm. **f** The NAS (NAFLD activity score) based on histological images was measured and found to be significantly decreased in the Foxo1^M-KO^ group (*n* = 6 mice/group). **g** Serum ALT and AST levels were decreased in the HFD-fed Foxo1^M-KO^ mice (IU/L) (*n* = 6 samples/group). **h** Quantitative PCR revealed that the levels of Srebp1c, Slc27a1, Fabp1, CD36, Fas, and Accα were significantly reduced, and the expression of Cpt1α, Acox1, and Acadm was increased in steatotic livers (*n* = 6 samples/group). *Notes:* Foxo1^M-KO^ significantly reduced the mRNA levels of genes responsible for fatty acid uptake and synthesis and increased fatty acid β-oxidation gene expression. All the data are presented as the mean±SD. Statistical analysis was performed using the permutation *t* test. *P < 0.05, **P < 0.01, ***P < 0.001, ****P < 0.0001.

To determine whether macrophage Foxo1 is involved in hepatic steatosis, we examined Foxo1 expression in Kupffer cells from steatotic livers. Indeed, wild-type (WT) mice fed an HFD displayed augmented JNK and NF-κB P65 phosphorylation (p-P65) in Kupffer cells (Fig. 1a). Interestingly, after 12 weeks of HFD feeding, nuclear Foxo1 expression substantially increased in Kupffer cells (Fig. 1a). This finding was confirmed by immunofluorescence staining, which showed that macrophage Foxo1 expression was increased in steatotic livers (Fig. 1b), suggesting that an HFD promotes Foxo1 activity, which is critically linked to NF-kB activation in hepatic macrophages. For determination of the role of macrophage Foxo1 in steatotic livers, Foxo1^M-KO^ and Foxo1^FL/FL^ mice were fed an HFD for 24 weeks. Foxo1^M-KO^ mice displayed lower liver-to-body weight ratios (Fig. 1c) and reduced levels of liver triglyceride (TG) and total cholesterol (TC) in liver tissue (Fig. 1d). Unlike the Foxo1^FL/FL^ controls, the Foxo1^M-KO^ mice exhibited alleviated hepatic steatosis with reduced hepatocyte ballooning and lipid accumulation in the liver (Fig. 1e, f). The serum levels of alanine aminotransferase (ALT) and aspartate aminotransferase (AST) were decreased in the HFD-fed Foxo1^M-KO^ mice (Fig. 1g). Foxo1^M-KO^ significantly reduced the mRNA levels of genes responsible for fatty acid uptake (fatty acid-binding protein 1 [Fabp1], fatty acid translocase [FAT/CD36] and solute carrier family 27 member 1 [Slc27a1]), and fatty acid synthesis (sterol regulatory element-binding protein-1c [Srebp1c], fatty acid synthase [Fas] and acetyl-CoA carboxylase α [Accα]) and increased gene expression related to fatty acid β-oxidation (carnitine palmitoyltransferase 1α [Cpt1α]), acyl-CoA oxidase 1 [Acox1] and acyl-CoA dehydrogenase medium chain [Acadm]) in the HFD-challenged livers (Fig. 1h). Moreover, Foxo1^M-KO^ significantly increased the expression of low-density lipoprotein receptor (Ldlr) but reduced the expression of macrophage scavenger receptor 1 (Msr1), which is involved in cholesterol uptake (Supplementary Fig. 2a). The mRNA levels of glucose metabolism-related genes showed no significant differences between Foxo1^FL/FL^ and Foxo1^M-KO^ groups in HFD-fed mice (Supplementary Fig. 2b). Similarly, the Foxo1^M-KO^ mice also exhibited reduced hepatic steatosis and inflammation in MCD diet-induced NASH (Supplementary Fig. 3). Collectively, these data indicate that macrophage Foxo1 plays a vital role in NASH development and progression.

### Macrophage Foxo1 deficiency inhibits STING-mediated liver inflammation and fibrosis in HFD-induced NASH

**Fig. 2:**
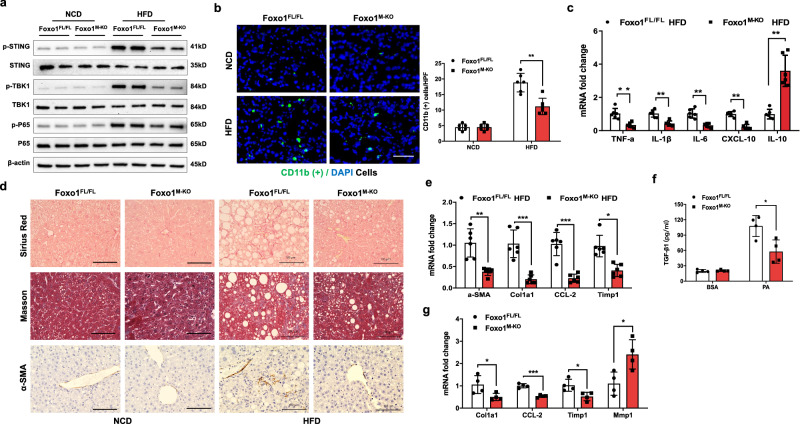
Macrophage Foxo1 deficiency inhibits STING-mediated liver inflammation and fibrosis in HFD-induced NASH. **a** The expression of p-STING, p-TBK1, and p-P65 was diminished in the livers of the Foxo1^M-KO^ mice after 24 weeks of HFD feeding. *Notes:* Foxo1^M-KO^ diminished p-STING, p-TBK1, and p-P65 expression in the HFD-challenged livers. **b** Immunofluorescence staining showed that CD11b^+^ macrophages were decreased in the livers of the Foxo1^M-KO^ mice after 24 weeks of HFD feeding (*n* = 6 mice/group). Quantification of CD11b^+^ macrophages; scale bars, 100 μm. **c** Quantitative RT‒PCR revealed that TNF-α, IL-1β, IL-6, and CXCL-10 levels were reduced in the Foxo1^M-KO^ mice after 24 weeks of HFD feeding, and IL-10 levels were increased in the Foxo1^M-KO^ mice (*n* = 6 samples/group). **d** Representative histological and immunohistochemical staining (Sirius Red, Masson, and α-SMA) showing reduced liver fibrosis in the livers of the HFD-challenged Foxo1^M-KO^ mice (*n* = 6 mice/group). Scale bars, 100 μm. **e** Quantitative RT‒PCR revealed that the expression of α-SMA, Col1a1, CCL2, and Timp1 was reduced in steatotic Foxo1^M-KO^ livers (*n* = 6 samples/group). **f** Hepatic Kupffer cells were stimulated with a mixture of 0.2 mM palmitic acid (PA) and 0.4 mM oleic acid (OA) for 24 h and then cocultured with primary hepatic stellate cells (HSCs). Compared with Foxo1^FL/FL^, Foxo1^M-KO^ markedly reduced TGF-β1 release from PA/OA-stimulated macrophages in the coculture supernatant (*n* = 4 samples/group). **g** Reduced HSC levels of the mRNAs encoding Col1α1, CCL2, Timp1, and augmented matrix metalloproteinase 1 (MMP1) after coculture were found (*n* = 4 samples/group). *Notes:* Foxo1^M-KO^ inhibits the mRNA expression of fibrogenic genes and reduces liver fibrosis in HFD-induced NASH. All the data are presented as the mean±SD. Statistical analysis was performed using the permutation *t* test. *P < 0.05, **P < 0.01, ***P < 0.001.

We then investigated whether macrophage Foxo1 influences STING activation and liver fibrosis in HFD-induced NASH. Indeed, after 24 weeks of HFD feeding, the expression of STING, phosphorylated TBK1 (p-TBK1), and p-P65 was augmented in the livers of the HFD-fed Foxo1^FL/FL^ mice (Fig. 2a). However, Foxo1^M-KO^ diminished p-STING, p-TBK1, and p-P65 expression in the HFD-challenged livers (Fig. 2a) and was accompanied by reduced CD11b^+^ macrophage accumulation (Fig. 2b). The mRNA levels of TNF-α, IL-1β, IL-6, and CXCL-10 in the livers of the HFD-fed Foxo1^M-KO^ mice were significantly lower than those in the Foxo1^FL/FL^ control mice, and the IL-10 level was elevated in the Foxo1^M-KO^ group (Fig. 2c). Notably, Foxo1^M-KO^ reduced liver fibrosis in HFD-challenged livers, as shown by Sirius red, Masson’s trichrome, and α-smooth muscle actin (αSMA) staining (Fig. 2d). Interestingly, the mRNA levels of fibrogenic genes, including αSMA, collagen type I α1 (Col1α1), chemokine (C-C motif) ligand 2 (CCL2), and tissue inhibitor of matrix metalloproteinase 1 (Timp1), were significantly decreased in the livers of the HFD-fed Foxo1^M-KO^ mice (Fig. 2e). In a macrophage/HSC coculture system, we found that Foxo1^M-KO^ markedly reduced TGF-β1 release from PA/OA-stimulated macrophages in the coculture supernatant compared to that of the Foxo1^FL/FL^ controls (Fig. 2f), accompanied by reduced HSC mRNA levels of Col1α1, CCL2, and Timp1 and augmented matrix metalloproteinase 1 (MMP1) expression after coculture (Fig. 2g). Taken together, these data suggest that macrophage Foxo1 signaling is crucial for the modulation of STING-mediated liver inflammation and fibrosis in HFD-induced NASH.

### Macrophage Foxo1 deficiency promotes the Notch1 and Hippo signaling pathways in response to HFD challenge

**Fig. 3:**
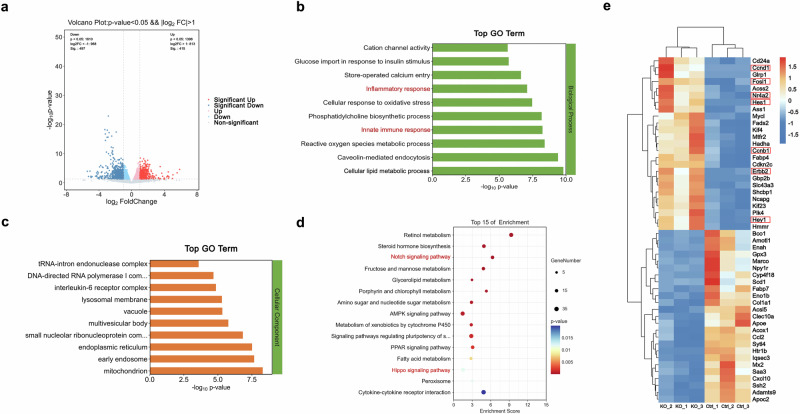
Macrophage Foxo1 deficiency promotes the Notch1 and Hippo signaling pathways in response to HFD challenge. Total RNA was extracted from liver macrophages from the Foxo1^FL/FL^ and Foxo1^M-KO^ mice after 24 weeks of HFD feeding. Deep RNA sequencing (RNA-seq) was subsequently performed. A modified Fisher’s exact test (enrichment score) was used for the functional enrichment analyses. Significantly upregulated or downregulated genes were determined by an adjusted *P* value of less than 0.05, which was set as the threshold to define DEGs, KEGG, and GO analysis. **a** The log2-fold changes in gene expression in the HFD-challenged liver macrophages from the Foxo1^M-KO^ mice compared to the Foxo1^FL/FL^ controls. Differentially expressed genes (DEGs) (*n* = 912, *P* < 0.05) in the HFD-challenged liver macrophages are indicated (red, upregulated, *n* = 415; green, downregulated, *n* = 497). **b**, **c** Gene Ontology (GO) enrichment analysis of cellular components and biological processes in the HFD-challenged liver macrophages from the Foxo1^M-KO^ mice. **d** Kyoto Encyclopedia of Genes and Genomes (KEGG) pathway enrichment analysis of transcripts differentially expressed in the HFD-challenged liver macrophages from the Foxo1^M-KO^ mice. **e** Heatmap showing the genes whose expression changed in the HFD-challenged liver macrophages from the Foxo1^M-KO^ mice. *Notes:* RNA-seq analysis revealed that the Foxo1^M-KO^ promotes the expression of genes related to the Notch1 and Hippo signaling pathways in the HFD-challenged macrophages.

We then tested whether macrophage Foxo1 affects gene expression and signaling pathways in response to HFD challenge. Liver macrophages were isolated from Foxo1^FL/FL^ and Foxo1^M-KO^ mice after 24 weeks of HFD feeding, and deep RNA sequencing (RNA-seq) analysis was performed. We found that Foxo1^M-KO^ significantly altered the expression of 912 genes (415 upregulated and 497 downregulated) in the HFD-challenged macrophages compared to the Foxo1^FL/FL^ controls (Fig. 3a). Gene Ontology (GO) analyses revealed that Foxo1^M-KO^ changed the expression of multiple genes related to the inflammatory response, innate immune response, cellular lipid metabolic process, etc. (Fig. 3b, c). Furthermore, Kyoto Encyclopedia of Genes and Genomes (KEGG) analyses revealed enrichment of differentially expressed genes related to the Notch signaling pathway, fatty acid metabolism, the PPAR signaling pathway, the AMPK signaling pathway, the Hippo signaling pathway, and cytokine‒cytokine receptor interactions (Fig. 3d, e). We found that Notch1 and Hippo signaling pathway-related genes, such as Hes1, Hey1, Fosl1, Ccnd1, Nr4a2, Ccnb1, and Erbb2, were significantly activated in the macrophages from the HFD-fed Foxo1^M-KO^ animals, which was further confirmed by quantitative RT‒PCR (Supplementary Fig. 5). These results indicate that the Notch1 and Hippo pathways play essential roles in regulating macrophage Foxo1-driven NASH progression.

### Macrophage Foxo1 deficiency increases YAP/NICD activity and inhibits STING activation in HFD-induced oxidative stress

**Fig. 4:**
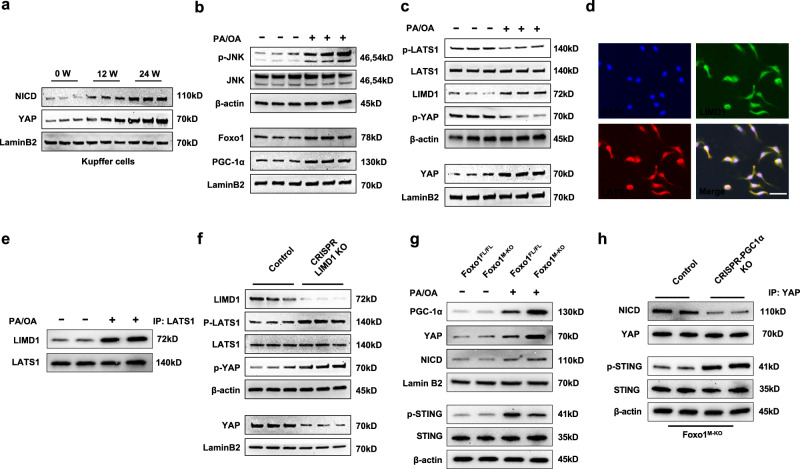
Macrophage Foxo1 deficiency increases YAP/NICD activity and inhibits STING activation in HFD-induced oxidative stress. **a** Nuclear YAP and NICD expression were substantially increased in macrophages after HFD feeding. The data are representative of three experiments. **b** Liver macrophages were isolated from WT mice and stimulated with a mixture of 0.2 mM palmitic acid (PA) and 0.4 mM oleic acid (OA) for 24 h. PA/OA stimulation activated JNK and increased nuclear Foxo1 and PGC-1α expression in macrophages. **c** PA and OA stimulation increased the expression of p-LATS1 and LIMD1, leading to reduced cytoplasmic YAP phosphorylation and increased nuclear YAP expression in macrophages. **d** Immunofluorescence staining showing macrophage LIMD1 (green) and LATS1 (red) colocalization in PA/OA-stimulated macrophages. DAPI was used to visualize nuclei (blue). Scale bars, 30 μm. **e** Immunoprecipitation analysis showed that PA/OA challenge augmented the colocalization and interaction of LIMD1 and LATS1 in macrophages. **f** Liver macrophages from WT mice were transfected with CRISPR/Cas9-mediated LIMD1 KO or control vector after PA/OA challenge. Moreover, LIMD1 KO increased cytoplasmic YAP phosphorylation and reduced nuclear YAP expression. **g** Disruption of macrophage Foxo1 markedly increased PGC-1α, YAP, and NICD levels and reduced p-STING expression in response to PA/OA challenge. **h** Liver macrophages from Foxo1^M-KO^ mice were transfected with CRISPR/Cas9-mediated PGC-1α KO or control vector after PA/OA stimulation. Immunoprecipitation analysis revealed that CRISPR/Cas9-mediated PGC-1α KO in the Foxo1^M-KO^ cells reduced the interaction of YAP with the NICD and augmented p-STING expression. *Notes:* Foxo1^M-KO^ activates YAP/NICD and inhibits STING activation in PA/OA-stimulated macrophages. All Western blots represent three experiments, and the data are presented as the mean±SD.

As the Hippo pathway and its effector protein YAP, a transcriptional coactivator, have been identified as important signaling cascades in regulating gene function, we then investigated whether HFD-induced oxidative stress affects YAP and the Notch1 intracellular domain (NICD) in the progression of NASH. To initially assess YAP and NICD function in HFD-challenged livers, we isolated liver macrophages from WT mice fed an HFD for 24 weeks. Indeed, after 12 and 24 weeks of HFD feeding, nuclear YAP and NICD expression substantially increased in macrophages (Fig. 4a). For determination of how HFD-induced oxidative stress modulates the Hippo–YAP pathway and Notch1 signaling, macrophages from WT mice were stimulated with a mixture of PA and OA for 24 h. Interestingly, PA and OA stimulation activated JNK and increased nuclear Foxo1 and PGC-1α expression in macrophages (Fig. 4b). Moreover, PA- and OA-induced oxidative stress-induced p-LATS1 and LIM domain-containing protein 1 (LIMD1) expression, leading to reduced cytoplasmic YAP phosphorylation and augmented nuclear YAP expression in macrophages (Fig. 4c). Notably, PA and OA challenge increased LIMD1 and LATS1 colocalization and interaction (Fig. 4d, e). However, CRISPR/Cas9-mediated LIMD1 knockout (KO) increased cytoplasmic YAP phosphorylation, which resulted in reduced nuclear YAP expression (Fig. 4f), suggesting that LIMD1 is essential for YAP activation in response to PA and OA stimulation. Surprisingly, disruption of macrophage Foxo1 markedly increased PGC-1α, YAP, and NICD expression and reduced p-STING expression in response to PA and OA challenge (Fig. 4g). To further elucidate the role of macrophage Foxo1 in the PCG-1α/YAP pathway under HFD-induced oxidative stress conditions, we used the antioxidant mitoTempo in Foxo1^M-KO^ macrophages after PA/OA stimulation. As expected, compared with the control treatment, MitoTempo treatment inhibited LATS1 and YAP phosphorylation but augmented nuclear PCG-1α and YAP expression in the PA/OA-stimulated Foxo1^M-KO^ macrophages (Supplementary Fig. 4a). Furthermore, CRISPR/Cas9-mediated PGC-1α KO in Foxo1^M-KO^ cells reduced the interaction of YAP with the NICD and augmented p-STING expression (Fig. 4h), suggesting that PGC-1α is a crucial mediator of the modulation of the YAP-NICD interaction and STING activation in HFD-induced oxidative stress.

### The YAP–NICD interaction targets cGAS and regulates STING-mediated inflammation

**Fig. 5:**
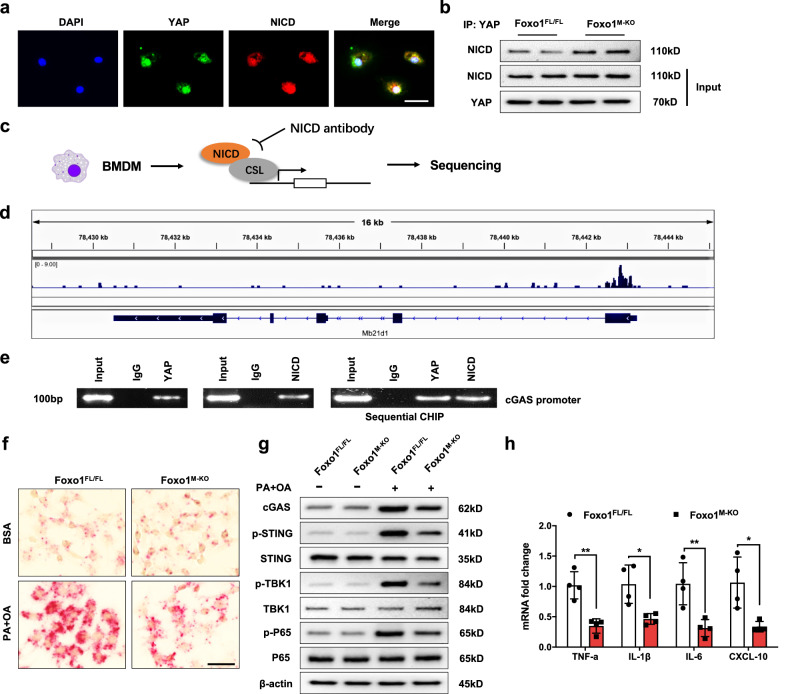
The YAP–NICD interaction targets cGAS and modulates STING-mediated inflammation. **a** Immunofluorescence staining showing the colocalization of YAP (green) and NICD (red) in the nuclei of macrophages after PA/OA stimulation. Scale bars, 30 μm. **b** Immunoprecipitation analysis showed that disruption of Foxo1 increased YAP binding to the NICD in Foxo1^M-KO^ but not in Foxo1^FL/FL^ macrophages. The data are representative of three experiments. **c** Experimental design of the NICD ChIP-seq analysis. BMMs were collected and fixed after culture with PA/OA for 24 h. Following chromatin shearing and NICD antibody selection, the precipitated DNA fragments bound by NICD-containing protein complexes were subjected to sequencing. **d** Localization of NICD-binding sites on the mouse *Mb21d1* (*cGAS*) gene. Five exons, 4 introns, the 3’ untranslated region (UTR), the 5’ UTR, and the transcription start site (TSS) of the mouse *cGAS* gene on chromosome 9 are shown. **e** ChIP‒PCR analysis of NICD and YAP binding to the *cGAS* promoter. Protein-bound chromatin was prepared from BMMs and immunoprecipitated with NICD or YAP antibodies. For sequential ChIP, the protein-bound chromatin was first immunoprecipitated with the NICD antibody, followed by elution with a second immunoprecipitation using the YAP antibody. Then, the immunoprecipitated DNA was analyzed by PCR. Normal IgG was used as a negative control. **f** RNA in situ hybridization assays showed that the transcript expression of the target gene cGAS was increased in the Foxo1^FL/FL^ macrophages after PA/OA stimulation (*n* = 4 samples/group). Scale bars, 50 μm. **g** Fatty acid stimulation increased cGAS, p-STING, p-TBK1, and p-P65 expression in the PA-stimulated Foxo1^FL/FL^ macrophages from the Foxo1^FL/FL^ or Foxo1^M-KO^ mice, whereas Foxo1 deletion diminished cGAS, p-STING, p-TBK1, and p-P65 expression. **h** qRT‒PCR analysis showed that TNF‒α, IL‒1β, IL‒6, and CXCL‒10 levels were decreased in the macrophages exposed to PA/OA (*n* = 4 samples/group). *Notes:* ChIP-seq analysis revealed that the YAP–NICD axis targets cGAS and regulates the STING-mediated inflammatory response in response to PA/OA stimulation. All western blots represent three experiments, and the data are presented as the mean±SD. Statistical analysis was performed using the permutation *t* test. *P < 0.05, **P < 0.01.

HFD-induced oxidative stress increased JNK-dependent Foxo1 transcriptional activity and LIMD1-mediated YAP nuclear translocation and activated Notch1 signaling, which prompted us to investigate the underlying molecular mechanism involved. Indeed, PA and OA stimulation induced the nuclear colocalization of YAP and NICD in bone marrow-derived macrophages (BMMs) from WT mice (Fig. 5a). Strikingly, disruption of Foxo1 increased YAP binding to the NICD in Foxo1^M-KO^ but not in Foxo1^FL/FL^ macrophages (Fig. 5b), suggesting that the YAP–NICD interaction plays a distinct role in the mechanism of macrophage Foxo1 signaling-mediated immune regulation in NASH progression. To determine how the YAP–NICD interaction regulates STING function, we performed chromatin immunoprecipitation (ChIP) coupled to massively parallel sequencing (ChIP-Seq) (Fig. 5c) (NCBI BioProject ID: PRJNA862184). Indeed, NICD ChIP-seq peaks were identified within the *Mb21d1* (*cGAS*) gene. One was located in the promoter region, and the others were located within introns or exons (Fig. 5d), indicating that the NICD was recruited to the promoter region of *Mb21d1* (*cGAS*). To validate the ChIP-seq peak in the cGAS promoter region, we performed ChIP-PCR using NICD and YAP antibodies in PA-treated BMMs. Primers were designed to detect the NICD DNA-binding site in the cGAS promoter. The sequential ChIP data revealed that YAP and the NICD were bound to the NICD-binding motif in the NICD-chromatin complex (Fig. 5e), confirming that YAP and the NICD are present in the same promoter region of cGAS. Thus, cGAS is a target gene regulated by the YAP–NICD complex. Moreover, an RNA in situ hybridization assay showed that the expression of the target gene cGAS was increased in Foxo1^FL/FL^ macrophages after fatty acid (PA and OA mixture) stimulation (Fig. 5f). In contrast, disruption of Foxo1 reduced cGAS transcript levels in the PA- and OA-stimulated Foxo1^M-KO^ macrophages (Fig. 5f). Furthermore, fatty acid stimulation increased cGAS, p-STING, p-TBK1, and p-P65 expression in the Foxo1^FL/FL^ macrophages (Fig. 5g), whereas Foxo1 deletion decreased cGAS, p-STING, p-TBK1, and p-P65 expression (Fig. 5g), accompanied by reduced TNF-α, IL-1β, IL-6, and CXCL-10 expression, in the PA-challenged macrophages (Fig. 5h). Taken together, these findings suggest that the macrophage YAP–NICD interaction is crucial for regulating the expression of the target gene cGAS and STING-mediated inflammation in HFD-induced oxidative stress.

### Disruption of macrophage Notch1 signaling activates cGAS and increases STING-mediated liver inflammation and fibrosis in HFD-induced NASH

**Fig. 6:**
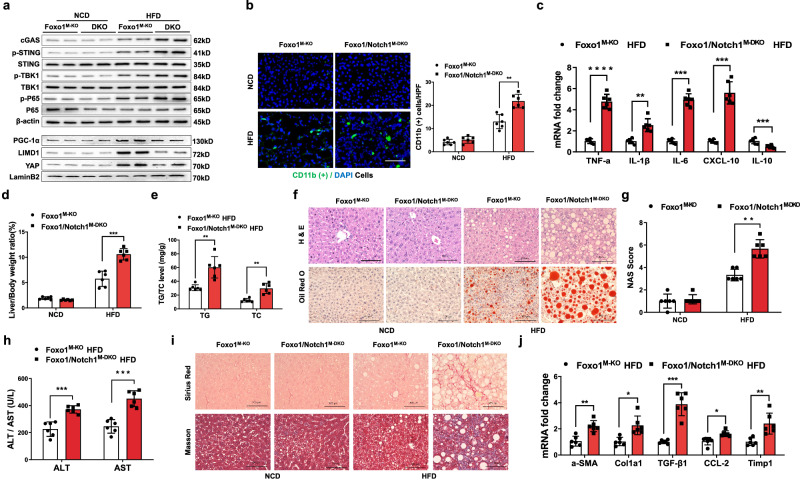
Disruption of macrophage Notch1 signaling activates cGAS and increases STING-mediated liver inflammation and fibrosis in HFD-induced NASH. **a** Foxo1/Notch1^M-DKO^ increased cGAS, p-STING, p-TBK1, p-P65, and nuclear PGC-1α, LIMD1, and YAP expression in steatotic livers after 24 weeks of HFD feeding. **b** Immunofluorescence staining showed that Foxo1/Notch1^M-DKO^ increased CD11b^+^ macrophage accumulation in steatotic livers (*n* = 6 mice/group). Quantification of CD11b^+^ macrophages; scale bars, 100 μm. **c** Foxo1/Notch1^M-DKO^ increased TNF-α, IL-1β, IL-6, and CXCL-10 expression and decreased IL-10 levels in steatotic livers (*n* = 6 samples/group). **d** The liver/body weight ratio was significantly increased in the HFD-fed Foxo1/Notch1^M-DKO^ mice (*n* = 6 samples/group). **e** TG and TC (mg/g) lipid levels were significantly increased in the HFD-fed Foxo1/Notch1^M-DKO^ mice (*n* = 6 samples/group). **f** Representative histological staining (H&E and Oil Red O) showing that the livers from the HFD-fed Foxo1/Notch1^M-DKO^ mice exhibited increased lipid accumulation (*n* = 6 mice/group). Scale bars, 100 μm. **g** NASs (NAFLD activity scores) were measured based on histological images and were significantly increased in the Foxo1/Notch1^M-DKO^ group (*n* = 6 mice/group). **h** Serum ALT and AST levels were increased in the HFD-fed Foxo1/Notch1^M-DKO^ mice (IU/L) (*n* = 6 samples/group). **i** Representative histological and immunohistochemical staining (Sirius Red and Masson) of steatotic liver tissues showing augmented liver fibrosis in the Foxo1/Notch1^M-DKO^ mice (*n* = 6 mice/group). Scale bars, 100 μm. **j** Increased mRNA expression of profibrotic genes, including αSMA, Col1α1, TGF-β1, CCL2, and TIMP1, in the Foxo1/Notch1^M-DKO^ livers after HFD feeding (*n* = 6 samples/group). *Notes:* Foxo1/Notch1^M-DKO^ activates cGAS, increases the STING-mediated inflammatory response, and exacerbates liver fibrosis in HFD-induced NASH. All the data are presented as the mean ± SD. Statistical analysis was performed using the permutation *t* test. *P < 0.05, **P < 0.01, ***P < 0.001, ****P < 0.0001.

To elucidate the mechanistic role of Notch activation in macrophage Foxo1 signaling-mediated immune regulation in NASH, we generated myeloid Foxo1 and Notch1 double-KO (Foxo1/Notch1^M-DKO^) mice. Indeed, we found that Foxo1/Notch1^M-DKO^ increased cGAS, p-STING, p-TBK1, p-P65, PGC-1α, LIMD1, and YAP expression in steatotic livers after 24 weeks of HFD feeding (Fig. 6a). Foxo1/Notch1^M-DKO^ increased CD11b^+^ macrophage accumulation (Fig. 6b) and TNF-α, IL-1β, IL-6, and CXCL-10 expression and decreased IL-10 levels (Fig. 6c) in the HFD-challenged livers. The liver-to-body weight ratios (Fig. 6d) and TG and TC levels (Fig. 6e) were significantly increased in the HFD-fed Foxo1/Notch1^M-DKO^ mice. The livers from the HFD-fed Foxo1/Notch1^M-DKO^ mice displayed increased lipid accumulation (Fig. 6f, g). In addition, Foxo1/Notch1^M-DKO^ increased the serum ALT and AST levels (Fig. 6h). In contrast to Foxo1^M-KO^, Foxo1/Notch1^M-DKO^ augmented liver fibrosis (Fig. 6i), with concomitant increases in the mRNA expression of profibrotic genes, including αSMA, Col1α1, TGF-β1, CCL2, and TIMP1, in the liver after HFD feeding (Fig. 6j). Taken together, these results suggest that Notch1 signaling is essential for the Foxo1-mediated regulation of hepatic steatosis, inflammation, and fibrosis in macrophages during NASH progression.

### YAP is required for macrophage Foxo1-mediated immune regulation of STING function in lipotoxicity-induced mitochondrial oxidative stress

**Fig. 7:**
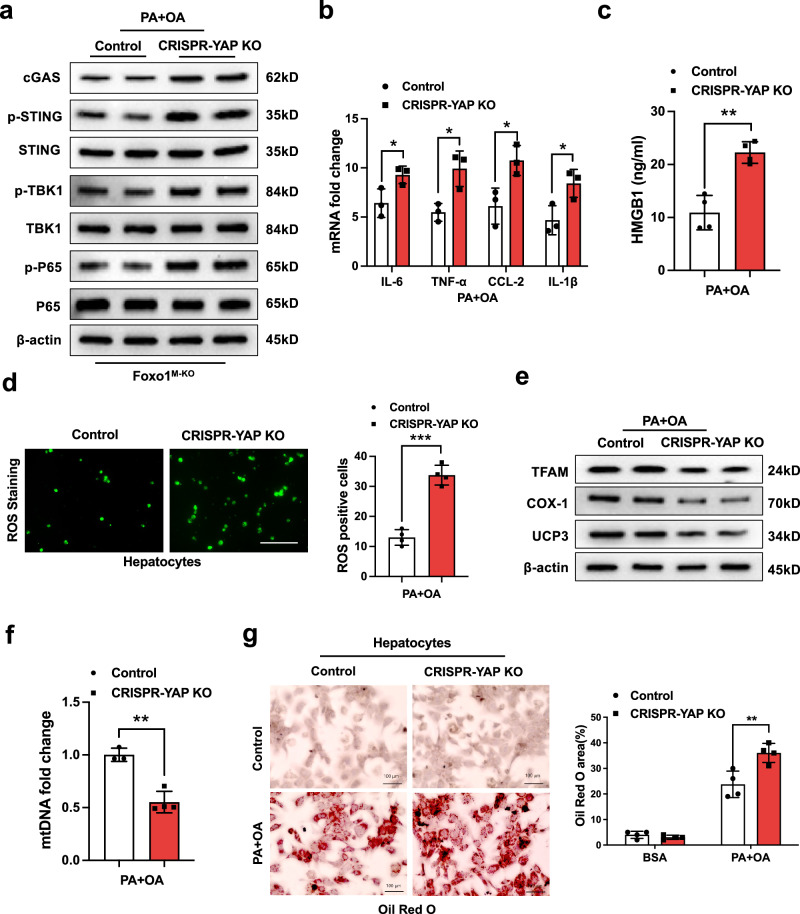
YAP is required for macrophage Foxo1-mediated immune regulation of STING function in lipotoxicity-induced mitochondrial oxidative stress. BMMs were isolated from Foxo1^M-KO^ mice, transfected with CRISPR/Cas9-mediated YAP knockout (p-CRISPR-YAP KO) or control vector, and then cocultured with primary hepatocytes after incubation with a 0.2 mM palmitic acid (PA) and 0.4 mM oleic acid (OA) mixture for 24 h. **a** CRISPR/Cas9-mediated YAP knockout augmented the expression of cGAS, p-STING, p-TBK1, and p-P65 in the PA-stimulated macrophages. The data are representative of three experiments. **b** The mRNA levels of IL-6, TNF-α, CCL2, and IL-β were elevated in the PA- and OA-stimulated Foxo1^M-KO^ macrophages. **c** ELISA analysis revealed that HMGB1 release was markedly increased in the p-CRISPR-YAP-KO cells but not in the control cells (*n* = 4 samples/group). **d** Immunofluorescence staining for ROS production showed that p-CRISPR-YAP KO in Foxo1^M-KO^ macrophages increased ROS production in hepatocytes after coculture and exposure to PA/OA (*n* = 4 samples/group). Quantification of ROS-producing macrophages (green). Scale bars, 100 μm. **e** The expression of TFAM, COX-1, and UCP3 was diminished in hepatocytes after coculture with p-CRISPR-YAP KO-transfected Foxo1^M-KO^ macrophages. The data are representative of three experiments. **f** Quantitative RT‒PCR analysis revealed that p-CRISPR-YAP KO reduced mtDNA levels in hepatocytes after coculture with p-CRISPR-YAP KO or control vector-transfected macrophages (*n* = 4 samples/group). *Notes:* YAP deletion in Foxo1^M-KO^ macrophages increased ROS production and reduced TFAM, Cox-1, UCP3, and mtDNA levels related to mitochondrial biogenesis in hepatocytes after coculture following PA/OA challenge. **g** Oil Red O staining revealed increased intracellular lipids in hepatocytes after coculture with the p-CRISPR-YAP-KO or control vector-transfected macrophages (*n* = 4 samples/group). Scale bars, 100 μm. All the data are presented as the mean±SD. Statistical analysis was performed using the permutation *t* test. *P < 0.05, **P< 0.01, ***P < 0.001.

Having demonstrated the pivotal role of the macrophage YAP–NICD interaction in modulating STING function in HFD-induced oxidative stress, we explored the mechanistic role of YAP in immune and metabolic regulation in vitro. Indeed, CRISPR/Cas9-mediated YAP knockout (p-CRISPR-YAP KO) augmented the expression of cGAS, p-STING, p-TBK1, and p-P65 (Fig. 7a) and the expression of mRNAs encoding IL-6, TNF-α, CCL2, and IL-1β (Fig. 7b) in the PA- and OA-stimulated Foxo1^M-KO^ macrophages. Under lipotoxic conditions, high mobility group box 1 (HMGB1) release was markedly increased in the p-CRISPR-YAP-KO cells but not in the control cells (Fig. 7c). Moreover, p-CRISPR-YAP KO in Foxo1^M-KO^ macrophages increased ROS production in hepatocytes after coculture with PA and OA (Fig. 7d). The expression of mitochondrial transcription factor A (TFAM), cytochrome c oxidase subunit I (COX-1) and uncoupling protein 3 (UCP3) was diminished in hepatocytes after coculture with p-CRISPR-YAP KO-transfected Foxo1^M-KO^ macrophages (Fig. 7e). Accordingly, p-CRISPR-YAP KO reduced mitochondrial DNA (mtDNA) levels (Fig. 7f) and augmented intracellular lipids in hepatocytes after PA and OA stimulation (Fig. 7g). To further elucidate the role of YAP in mitochondrial biogenesis under HFD-induced oxidative stress conditions, we used the antioxidant mitoTempo in a coculture of hepatocytes/YAP-deficient macrophages after PA/OA challenge. Interestingly, mitoTempo treatment restored TFAM and mtDNA levels in hepatocytes after coculture with YAP-deficient macrophages in response to PA/OA stimulation (Supplementary Fig. 4b, c). Overall, YAP functions as a transcriptional coactivator of the NICD in regulating STING function in response to lipotoxicity-induced mitochondrial oxidative stress.

### The Foxo1–YAP axis modulates STING-mediated liver inflammation and steatosis in HFD-induced NASH

**Fig. 8:**
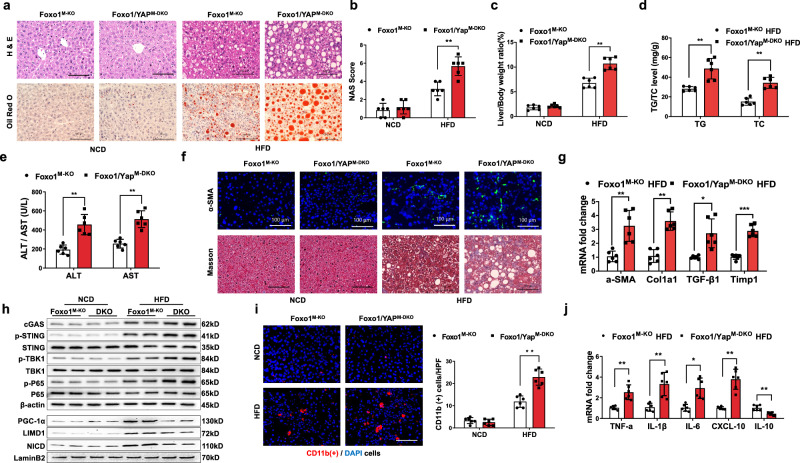
The Foxo1–YAP axis modulates STING-mediated liver inflammation and steatosis in HFD-induced NASH. **a** Representative histological staining (H&E and Oil Red O) showing that the Foxo1^M-KO^ mice exhibited decreased lipid accumulation, whereas the Foxo1/YAP^M-DKO^ mice exhibited increased hepatic steatosis after 24 weeks of HFD feeding (*n* = 6 mice/group). Scale bars, 100 μm. **b** The NAS (NAFLD activity score) based on histological images was measured and found to be significantly increased in the Foxo1/YAP^M-DKO^ group (*n* = 6 mice/group). **c** The liver/body weight ratios were significantly greater in the Foxo1/YAP^M-DKO^ mice (*n* = 6 samples/group). **d** The TG and TC levels (mg/g) were significantly increased in the Foxo1/YAP^M-DKO^ mice (*n* = 6 samples/group). **e** The Foxo1/YAP^M-DKO^ mice exhibited significantly increased serum ALT and AST levels (IU/L) (*n* = 6 samples/group). **f** Representative immunofluorescence and immunohistochemistry images (α-SMA and Masson) showing significantly increased liver fibrosis in the Foxo1/YAP^M-DKO^ livers (*n* = 6 mice/group). Scale bars, 100 μm. **g** Quantitative RT‒PCR analysis showed that Foxo1/YAP^M-DKO^ increased cGAS, p-STING, p-TBK1, and p-P65 expression in steatotic livers (*n* = 6 samples/group). **h** Western blot analysis revealed that Foxo1/YAP^M-DKO^ increased cGAS, p-STING, p-TBK1, and p-P65 expression and increased nuclear PGC-1α, LIMD1, and NICD expression in steatotic livers. The data are representative of three experiments. **i** Immunofluorescence staining showed increased CD11b^+^ macrophage accumulation in ischemic livers (*n* = 6 mice/group). Quantification of CD11b^+^ macrophages; scale bars, 100 μm. **j** The mRNA levels of TNF-α, IL-1β, IL-6, and CXCL-10 were increased, and the IL-10 level was decreased in the steatotic Foxo1/YAP^M-DKO^ livers (*n* = 6 samples/group). *Notes:* Foxo1/YAP^M-DKO^ exacerbates STING-mediated liver inflammation, steatosis, and fibrosis in mice with HFD-induced NASH. All the data are presented as the mean±SD. Statistical analysis was performed using the permutation *t* test. *P < 0.05, **P < 0.01, ***P < 0.001.

Next, we analyzed the role of the Foxo1–YAP axis in modulating lipid metabolism and STING-mediated inflammation in NASH. Using the generated myeloid Foxo1 and YAP double-KO (Foxo1/YAP^M-DKO^) mice, we found that Foxo1^M-KO^ decreased lipid accumulation, whereas Foxo1/YAP^M-DKO^ resulted in increased hepatic steatosis after 24 weeks of HFD feeding (Fig. 8a, b). Consistent with these results, the liver-to-body weight ratios (Fig. 8c) and TG and TC levels (Fig. 8d) were significantly increased in the Foxo1/YAP^M-DKO^ mice. Compared with the WT mice, the Foxo1/YAP^M-DKO^ mice exhibited significantly increased serum ALT levels (Fig. 8e), liver fibrosis (Fig. 8f), and mRNA levels of αSMA, Col1α1, TGF-β1, CCL2, and TIMP1 in the HFD-challenged livers (Fig. 8g). Notably, Foxo1/YAP^M-DKO^ increased cGAS, p-STING, p-TBK1, p-P65, PGC-1α, LIMD1 and NICD expression (Fig. 8h), which was accompanied by increased CD11b^+^ macrophage accumulation (Fig. 8i) and TNF-α, IL-1β, IL-6, and CXCL-10 expression and decreased IL-10 levels (Fig. 8j) in the HFD-challenged livers. Hence, these findings indicate the essential role of YAP in modulating STING-mediated hepatic steatosis, inflammation, and fibrosis in NASH.

## Discussion

Accumulating evidence suggests that triggers of hepatic inflammation are critical for the development of NASH and that inflammation activates HSCs, leading to different degrees of fibrosis^23^. Lipid-induced hepatocellular injury and oxidative stress play central roles in the activation of hepatic Kupffer cells to trigger innate immunity during NASH progression^24^. As a key component of innate immunity, macrophages can release proinflammatory mediators to exacerbate hepatic inflammation and fat metabolic dysfunction^24^. Thus, immune and inflammatory pathways have emerged as critical players contributing to the development of NASH. Here, we reported that HFD-induced oxidative stress-activated Foxo1, the Hippo–YAP pathway, and Notch1 signaling in liver macrophages. Disruption of macrophage Foxo1 increased PCG-1α, promoted the YAP–NICD interaction, and ameliorated hepatic steatosis and inflammation in NASH patients. Notably, we showed that macrophage Foxo1 cooperated with the YAP and Notch1 signaling pathways to control NASH progression by modulating cGAS-STING-mediated innate immune responses (Supplementary Fig. 1).

The Foxo1 transcription factor has multiple cell functions in modulating innate immunity in response to oxidative stress^19^. Foxo1 was shown to promote macrophage-mediated inflammation by activating innate TLR4 signaling^13^. Our previous studies revealed that disruption of Foxo1 inhibited NLRP3 activation and alleviated liver inflammatory injury^19^. Foxo1 modulates macrophage function via metabolic reprogramming^25^. Current studies have shown that an HFD increases Foxo1 activity in liver macrophages from steatotic livers, whereas disruption of macrophage Foxo1 reduces lipid synthesis-related gene expression and increases cholesterol and fatty acid metabolism. Indeed, an HFD activated the JNK pathway, leading to increased Foxo1 translocation from the cytoplasm to the nucleus, indicating that JNK is required for Foxo1 translocation in response to lipotoxicity-induced oxidative stress. Interestingly, HFD-induced oxidative stress activated STING signaling, whereas macrophage Foxo1 deficiency inhibited STING function and was accompanied by reduced hepatic steatosis, fibrosis, and inflammation in steatotic livers. These results indicate that macrophage Foxo1 signaling is essential for controlling STING-mediated innate immune responses during NASH progression.

We also found that macrophage Foxo1 deficiency promoted the Hippo–YAP pathway and Notch1 signaling in liver macrophages from steatotic livers. Metabolic stress upregulated LIMD1, a multifunctional adaptor protein^26^. LIMD1 directly interacts with LATS1 kinase, which modulates YAP activity in macrophages after PA and OA treatment. However, disruption of LIMD1 resulted in reduced YAP activity in PA and OA-stimulated macrophages, suggesting that LIMD1 is essential for YAP activation. Previous reports have demonstrated that deletion of Foxo1 can restore mitochondrial biogenesis^27^ and that PINK1 is a key player in mitochondrial homeostasis under oxidative stress conditions^28^. PINK1 deficiency induces a STING-dependent inflammatory response^29^. Consistent with these results, we found that macrophage Foxo1 deficiency reduced ROS production, promoted PINK1, and inhibited STING activation in Foxo1^M-KO^ macrophages in response to fatty acid stimulation (Supplementary Fig. 6). Moreover, we found that macrophage Foxo1 deficiency activated PGC-1α but inhibited STING in response to fatty acid stimulation. Indeed, as a transcriptional coactivator, PGC-1*α* is linked to metabolic regulation, redox control, and inflammatory pathways^30^. Increased PGC-1α reduces oxidative stress and proinflammatory mediators in many metabolic disorders^31^. Interestingly, fatty acid stimulation induced the interaction between YAP and the NICD in Foxo1-deficient macrophages. Disruption of PGC-1α inhibited the interaction of YAP with the NICD and increased STING activation. These results suggest that macrophage Foxo1 signaling-mediated PGC-1α is crucial for regulating the YAP–NICD axis-mediated STING activation during HFD-induced oxidative stress.

The mechanisms underlying Foxo1-mediated regulation of the YAP–NICD axis by selectively influencing the STING function in steatotic livers were investigated. HFD feeding induced oxidative stress and activated JNK, promoting the Hippo–YAP and Notch1 signaling pathways, suggesting the importance of the Hippo–YAP and Notch1 pathways in STING function. As expected, our in vitro study revealed that macrophage YAP and NICD colocalized in the nucleus and increased the nuclear expression of YAP and NICD in response to PA and OA stimulation. Notably, the NICD interacted with YAP via direct binding. Thus, we speculate that the YAP–NICD interaction is essential for the modulation of STING function in NASH progression. This finding was further supported by the ChIP and ChIP-sequencing data, which showed that NICD colocalized with YAP on the promoter of cGAS, indicating that cGAS is a target gene of the NICD regulated by the YAP and NICD complex. Moreover, in a macrophage/hepatocyte or HSC coculture system, fatty acid stimulation activated the macrophage cGAS-STING pathway. Disruption of cGAS reduced STING-mediated inflammatory responses, lipid accumulation, and fibrogenic gene expression. These results suggest that the YAP–NICD axis modulates liver inflammation, steatosis, and fibrosis by targeting the cGAS-STING pathway in NASH.

We demonstrated that PA- and OA-induced oxidative stress activated the Hippo–YAP pathway in macrophages. Disruption of YAP increases the innate immune response, whereas activation of YAP orchestrates the immunosuppressive response following tissue injury^17,18^. Consistent with these findings, YAP deficiency promoted cGAS/STING activation and inflammatory responses upon exposure to PA/OA challenge. Notably, macrophage YAP deficiency increased HMGB1 release and augmented hepatocyte ROS production after fatty acid stimulation. Indeed, HMGB1 is a widely expressed protein that acts as a danger signal in triggering oxidative stress^32^. Increased ROS production induces mitochondrial dysfunction, particularly mtDNA damage^33^. We found that disruption of macrophage YAP reduced hepatocyte mtDNA levels after coculture in response to PA and OA stimulation. As a transcription factor, TFAM is crucial for activating mitochondrial DNA transcription and biogenesis^34^. Moreover, TFAM regulates lipid metabolism and confers protection against HFD-induced obesity^35^. Our current study revealed that deletion of macrophage YAP diminished TFAM expression and was accompanied by increased fatty acid-induced lipid accumulation in hepatocytes. These findings indicate that macrophage YAP is essential for modulating STING-mediated inflammation, lipid metabolism, and mitochondrial biogenesis.

Notably, YAP was revealed to act as a transcriptional coactivator of the NICD in macrophage Foxo1-mediated immune regulation of cGAS/STING function during NASH progression. Deletion of YAP in the Foxo1^M-KO^ mice exacerbated HFD-induced hepatic steatosis, fibrosis, and inflammation and increased cGAS-STING-mediated innate immune responses. Indeed, long-term HFD feeding increased oxidative stress and induced mitochondrial dysfunction, leading to mtDNA release and the induction of the cGAS-STING-mediated innate immune signaling cascade. The YAP–NICD interaction modulates STING function, suggesting that the YAP–NICD axis is essential for regulating liver inflammation and lipid metabolism. Our previous studies demonstrated the importance of Notch1 signaling in regulating TLR4- or NLRP3-driven inflammatory responses^20,36^. Indeed, Notch signaling is activated by dual proteolytic cleavage, which releases its intracellular domain (NICD), which binds to the nuclear recombinant recognition sequence binding protein at the Jκ site (RBP-J) to induce the expression of Notch target genes^37^. Consistent with these findings, our results showed that disruption of Notch1 signaling in Foxo1-deficient livers increased cGAS and STING activation, leading to exacerbated hepatic steatosis, fibrosis, and inflammation. Therefore, our findings revealed the key role of the YAP–NICD axis in modulating the cGAS-STING innate immune pathway during NASH development.

In conclusion, we identified a previously unrecognized role of the macrophage Foxo1–YAP–NICD axis in controlling STING-mediated innate immune responses in steatotic livers. The functional interplay between the Hippo–YAP pathway and Notch1 signaling is crucial for regulating STING function in NASH progression, suggesting potential therapeutic targets for NASH.

## Supplementary information

Supplementary Materials
