# Supplementary material for: The Foxo1-YAP-Notch1 axis reprograms STING-mediated innate immunity in NASH progression
Source: Exp Mol Med. 2024 Aug 9;56(8):1843–55. doi: 10.1038/s12276-024-01280-5 (PMC11372114; doi:10.1038/s12276-024-01280-5)
Supplement: Supplementary file 1 — Supplementary Materials [file 12276_2024_1280_MOESM1_ESM.pdf]

Supplementary Figures

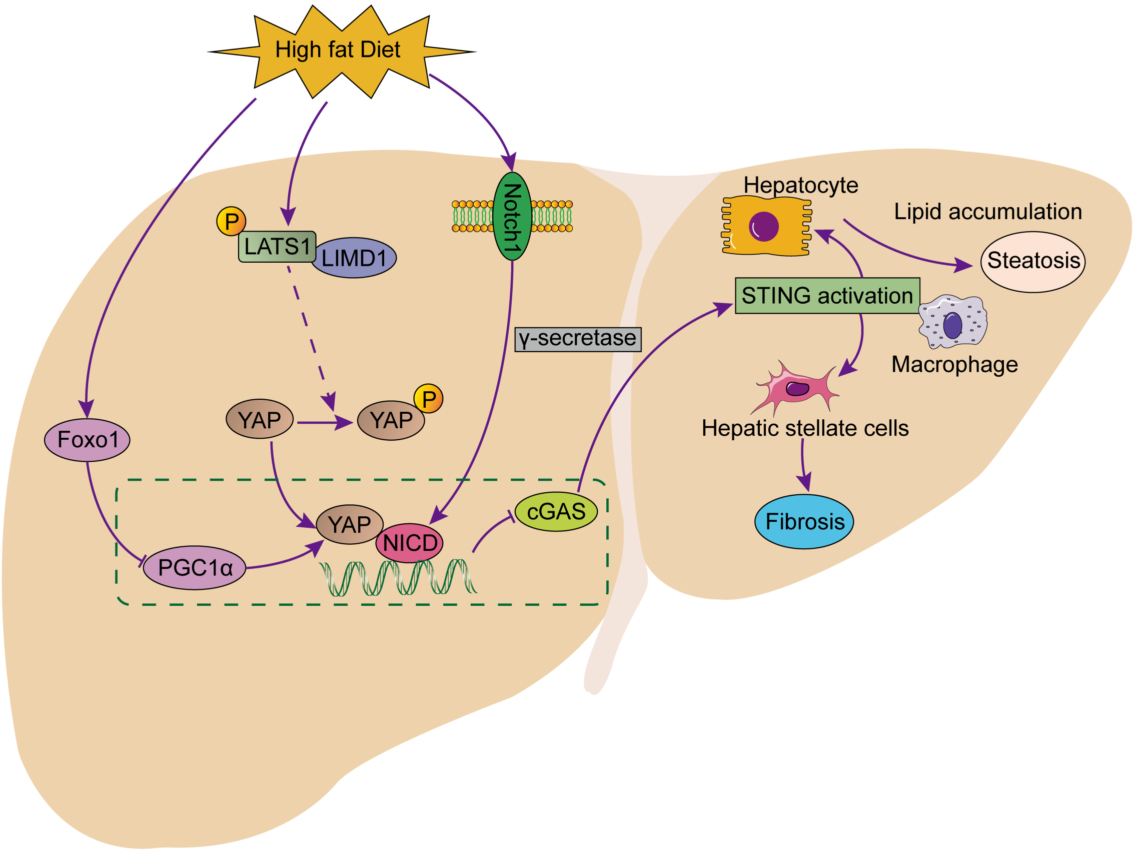

Supplementary Fig. 1. Schematic illustration of molecular mechanisms of macrophage Foxo1-YAP-Notch1 axis in regulating STING-mediated innate immunity in NASH progression. High-fat diet (HFD)-induced oxidative stress activates macrophage Foxo1, YAP, and Notch1 signaling. Macrophage Foxo1 deficiency enhances YAP activity and regulates cholesterol/fatty acid synthesis and mitochondrial functions by activating PGC-1α-mediated YAP activity. YAP directly interacts with NICD, modulating its target gene *Mb21d1* (cGAS) and downstream effector STING, leading to reduced liver steatosis and inflammation.

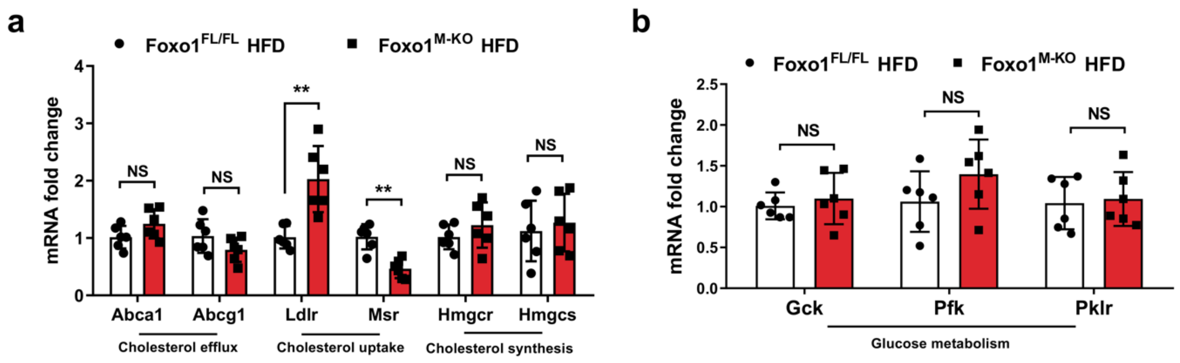

Supplementary Fig. 2. Macrophage Foxo1 regulates cholesterol and glucose metabolism. (a) Quantitative PCR analysis of gene expression related to cholesterol efflux (Abca1, Abcg1) and cholesterol synthesis (Hmgcr, Hmgcs). The mRNA levels of Abca1, Abcg1, Hmgcr, and Hmgcs showed no significant differences between Foxo1$^{FL/FL}$ and Foxo1$^{M\text{-}KO}$ groups in HFD-fed mice. The levels of Ldlr were increased, and Msr1 levels were decreased, which reflected enhanced cholesterol uptake in Foxo1$^{M\text{-}KO}$ mice. (n=6 samples/group). (b) Quantitative PCR analysis of gene expression related to glucose metabolism. The mRNA levels of glucose metabolism-related genes (Gck, Pfk, Pklr) showed no significant differences between Foxo1$^{FL/FL}$ and Foxo1$^{M\text{-}KO}$ groups in HFD-fed mice. All data represent the mean±SD. Statistical analysis was performed using the Permutation t-test. **p<0.01.

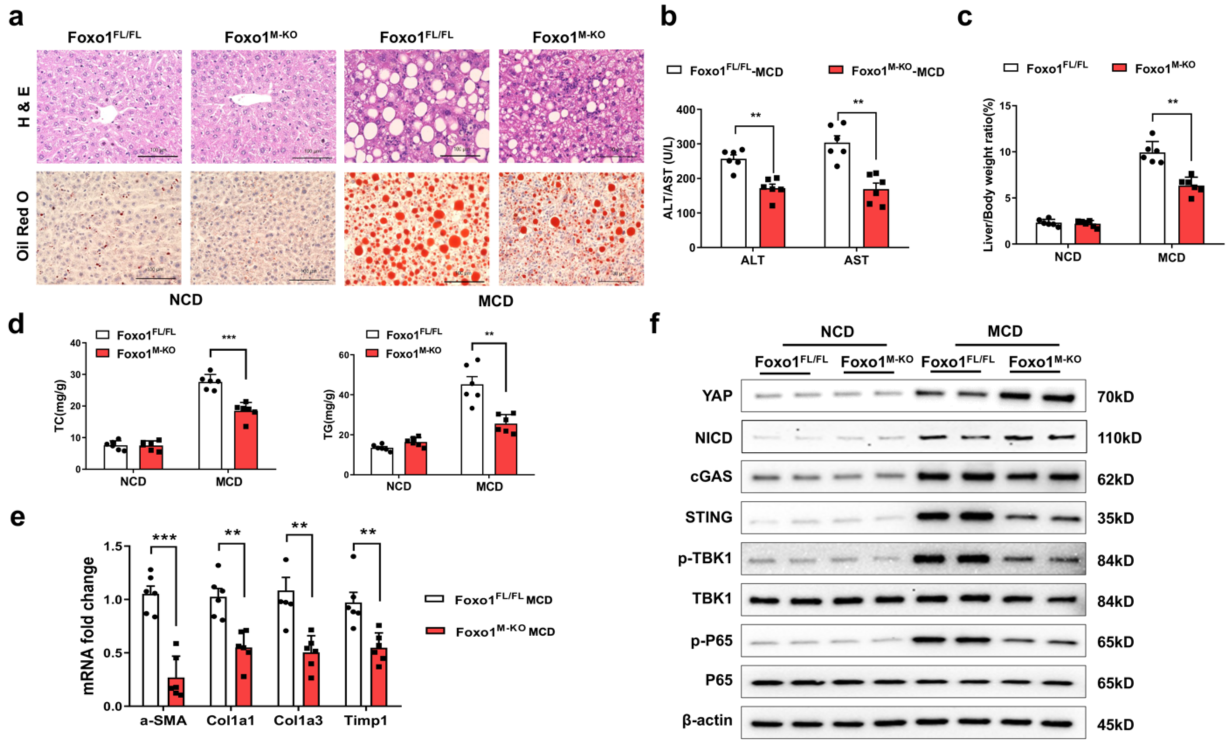

Supplementary Fig. 3. Macrophage Foxo1 deficiency reduces hepatic steatosis and inflammation in MCD-induced NASH. (a) Representative histological staining (H&E and Oil Red O) displayed that Foxo1$^{M\text{-}KO}$ mice alleviated hepatic steatosis with reduced hepatocyte ballooning and lipid accumulation in the livers (n=6 mice/group). Scale bars, 100μm. (b) Serum ALT and AST levels were decreased in MCD-fed Foxo1$^{M\text{-}KO}$ mice (IU/L) (n=6 samples/group). (c) Foxo1$^{M\text{-}KO}$ mice displayed lower liver-to-body weight ratios (n=6 samples/group). (d) Levels of liver TG and TC (mg/g) in Foxo1$^{M\text{-}KO}$ livers were reduced (n=6 samples/group). (e) mRNA levels of a-SMA, Col1a1, Col1a3, Timp1 were significantly reduced in steatotic livers (n=6 samples/group). (f) The protein expression of YAP and NICD were upregulated in MCD-fed Foxo1$^{M\text{-}KO}$ livers, along with decreased cGAS, STING, p-TBK1, and p-P65 expression. Representative of three experiments. All data represent the mean±SD. Statistical analysis was performed using the Permutation t-test. **p<0.01, ***p<0.001.

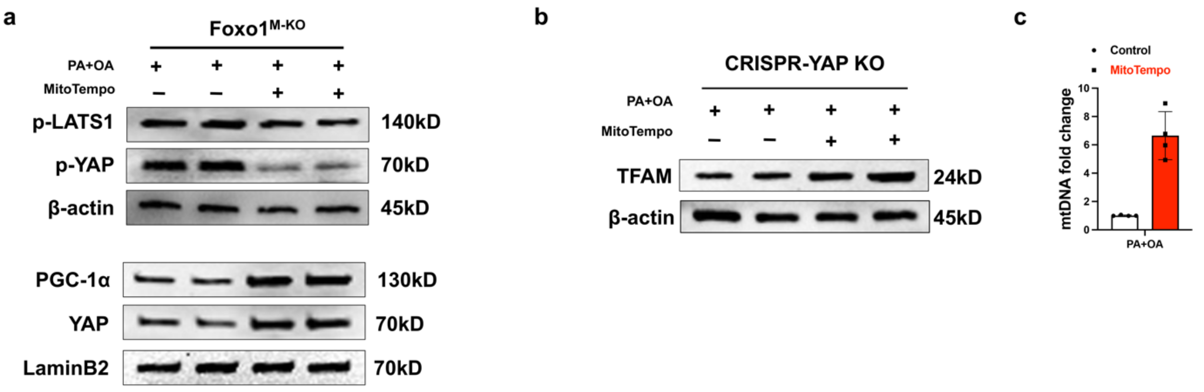

Supplementary Fig. 4. Mitochondrial stress plays a vital role in Foxo1-induced NASH progression. BMMs were isolated from the Foxo1$^{M\text{-}KO}$ mice and transfected with CRISPR/Cas9-mediated YAP knockout (p-CRISPR-YAP KO) or control vector and then co-cultured with primary hepatocytes after incubation with 0.2 mM palmitic acid (PA) and 0.4 mM oleic acid (OA) mixture for 24h. BMMs were pre-treated with or without MitoTempo (500μM, 1h). (a) MitoTempo treatment inhibited the level of p-LATS1 and p-YAP while augmenting nuclear PGC-1α and YAP expression in PA/OA-stimulated macrophages. Representative of three experiments. (b) The expression of TFAM was restored in hepatocytes after co-culture with MitoTempo pre-treated p-CRISPR-YAP KO-transfected macrophages. Representative of three experiments. (c) Quantitative RT-PCR analysis revealed that MitoTempo pre-treatment restored mtDNA levels in hepatocytes after co-culture with p-CRISPR-YAP KO macrophages. (n=4 samples/group). All data represent the mean±SD. Statistical analysis was performed using the Permutation t-test. *p<0.05.

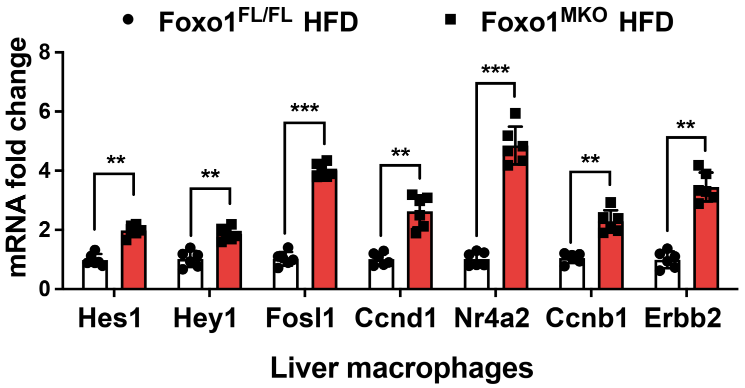

Supplementary Fig. 5. Confirmation of the Notch1 and Hippo signaling pathways related genes. Quantitative PCR analysis of mRNA expression of the Notch1 and Hippo signaling pathways related genes (Hes1, Hey1, Fosl1, Ccnd1, Nr4a2, Ccnb1, Erbb2) in HFD-challenged liver macrophages from the $Foxo1^{FL/FL}$ and $Foxo1^{M\text{-}KO}$ mice (n=6 samples/group). All data represent the mean±SD. Statistical analysis was performed using the Permutation t-test. **p<0.01, ***p<0.001.

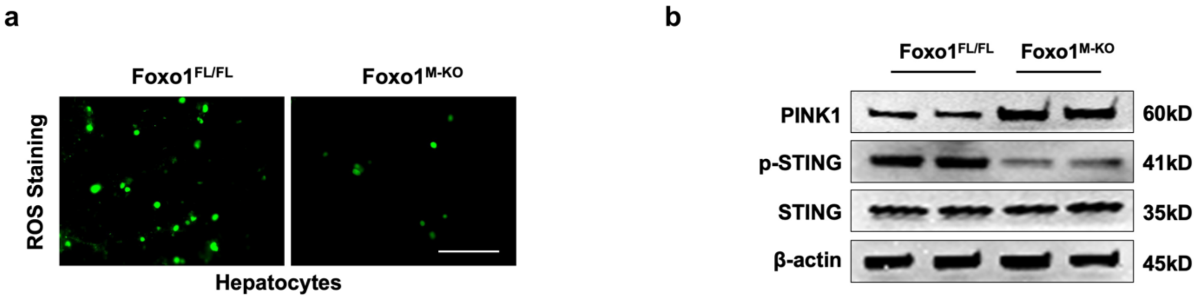

Supplementary Fig. 6. Macrophage Foxo1 deficiency reduces ROS-induced oxidative stress, promotes PNIK1, and inhibits STING activation in PA/OA-stimulated macrophages. BMMs were isolated from the $Foxo1^{FL/FL}$ and $Foxo1^{M\text{-}KO}$ mice and then co-cultured with primary hepatocytes after incubation with 0.2 mM palmitic acid (PA) and 0.4 mM oleic acid (OA) mixture for 24h. (a) Immunofluorescence staining for ROS production showed that $Foxo1^{M\text{-}KO}$ decreased ROS production in hepatocytes after co-culture with macrophages exposed to PA and OA challenge, compared with $Foxo1^{FL/FL}$ controls (n=4 samples/group). Quantification of ROS-producing macrophages (green). Scale bars, 100 μm. (b) The protein expression of PNIK1 was augmented while p-STING was diminished in hepatocytes after co-culture with $Foxo1^{M\text{-}KO}$ macrophages compared to the $Foxo1^{FL/FL}$ controls. Representative of three experiments.

Supplementary Table 1: Primers used in qRT-PCR studies.

Target genes	Forward primers	Reverse primers
HPRT	5’- TCAACGGGGGACATAAAAGT -3’	5’- TGCATTGTTTTACCAGTGTCAA -3’
TNF-α	5’- GCCTCTTCTCATTCCTGCTTGT -3’	5’- GATGATCTGAGTGTGAGGGTCTG -3’
IL-6	5’- GCTACCAAACTGGATATAATCAGGA -3’	5’- CCAGGTAGCTATGGTACTCCAGAA -3’
IL-1β	5’- TGTAATGAAAGACGGCACACC -3’	5’- TCTTCTTTGGGTATTGCTTGG -3’
CXCL-10	5’- CCAAGTGCTGCCGTCATTTTC -3’	5’- GGCTCGCAGGGATGATTTCAA -3’
a-SMA	5’-GTCCCAGACATCAGGGAGTAA -3’	5’- TCGGATACTTCAGCGTCAGGA -3’
CCL-2	5'- GAAGGAATGGGTCCAGACAT -3'	5'- ACGGGTCAACTTCACATTCA -3'
Col1a1	5’- GACATGTTCAGCTTTGTGGAC -3’	5’- GCAGCTGACTTCAGGGATG -3’
Timp1	5’- GGCATCCTCTTGTTGCTATCACTG -3’	5’- GTCATCTTGATCTCATAACGCTGG -3’
Mmp1	5’- AACTACATTTAGGGGAGAGGTGT -3’	5’- GCAGCGTCAAGTTTAACTGGAA -3’
TGF-β1	5’- CTCCCGTGGCTTCTAGTGC -3’	5’- GCCTTAGTTTGGACAGGATCTG -3’
Fas	5’- CTGCGGAAACTTCAGGAAATG -3’	5’- GGTTCGGAATGCTATCCAGG -3’
Srebp1c	5’- CACTTCTGGAGACATCGCAAAC -3’	5’- ATGGTAGACAACAGCCGCATC -3’
Fabp1	5’- ATGAACTTCTCCGGCAAGTACC -3’	5’- CTGACACCCCCTTGATGTCC -3’
CD36	5’- AGATGACGTGGCAAAGAACAG -3’	5’- CCTTGGCTAGATAACGAACTCTG -3’
Acca	5’- ATGGGCGGAATGGTCTCTTTC -3’	5’- TGGGGACCTTGTCTTCATCAT -3’
Cpt1a	5’- AGGACCCTGAGGCATCTATT -3’	5’- ATGACCTCCTGGCATTCTCC -3’
Slc27a1	5'-TGCACAGCAGGTACTACCGCAT -3'	5'-TGCGCAGTACCACCGTCAAC-3'
Acox1	5'-GTCTCCGTCATGAATCCCGA-3'	5'-TGCGATGCCAAATTCCCTCA-3'
Acadm	5'-AAACATGGGCCAGCGATGCTCT-3'	5'-AGGGCATACTTCGTGGCTTCGT-3'
Abca1	5'-ATTGCCAGACGGAGCCG-3'	5'-TGCCAAAGGGTGGCACA-3'
Abcg1	5'-TTCGCTGCTCTGGGTACCA-3'	5'-TGTCACGGGACCCACAAAT-3'
Ldlr	5'-GCGGCTTCCGGTTGGT-3'	5'-AGAGCTGGCTGCAGGTGTCT-3'
Msr1	5'-GAAATTTGACGCACGTTCAATG-3'	5'-TTTTTAGTGCTGTGAGGAAGGGAT-3'
Hmgcr	5'-CCAAACCCCGTAACCCAAA-3'	5'-CGACTATGAGCGTGAACAAGGA-3'
Hmgcs	5'-TCTTGGGATGGACGATATGCT-3'	5'-GGCATTTCCTGTGGCATATATAGC-3'
Gck	5'-AGACGAAACACCAGATGTATTCC-3’	5'-GAAGCCCTTGGTCCAGTTGAG-3’
Pfk	5'-GAACTACGCACACTTGACCAT-3’	5'-CTCCAAAACAAAGGTCCTCTGG-3’
Pklr	5’- GAACATTGCACGACTCAACTTC -3’	5’- CAGTGCGTATCTCGGGACC -3’
Hes1	5’- CCAGCCAGTGTCAACACGA -3’	5’- AATGCCGGGAGCTATCTTTCT -3’
Hey1	5’- GCGCGGACGAGAATGGAAA -3’	5’- TCAGGTGATCCACAGTCATCTG -3’
Fosl1	5’- ATGTACCGAGACTACGGGGAA -3’	5’- CTGCTGCTGTCGATGCTTG -3’
Ccnd1	5’- GCGTACCCTGACACCAATCTC -3’	5’- CTCCTCTTCGCACTTCTGCTC -3’

Nr4a2	5’- GTGTTCAGGCGCAGTATGG -3’	5’- TGGCAGTAATTTCAGTGTTGGT -3’
Ccnb1	5’- AAGGTGCCTGTGTGTGAACC -3’	5’- GTCAGCCCCATCATCTGCG -3’
Erbb2	5’- ACCGACATGAAGTTGCGACTC -3’	5’- AGGTAAGCTCCAAATTGCCCT -3’
cGAS-promoter	5’- TTCTGCAAAGTAGGCAGCGT -3’	5’- AACTTGTCTAACAAGCATTCGCT -3’
mtDNA (Mouse)	5’- CGCCTACTCCTCAGTTAGCCA -3’	5’- TGATGTGAGGCCATGTGCGA -3’
GAPDH	5’- ATGGGACGATGCTGGTACTGA -3’	5’- TGCTGACAACCTTGAGTGAAAT -3’
